# Nucleotide-binding mechanisms in pseudokinases

**DOI:** 10.1042/BSR20150226

**Published:** 2016-01-15

**Authors:** Henrik M. Hammarén, Anniina T. Virtanen, Olli Silvennoinen

**Affiliations:** * School of Medicine, University of Tampere, Biokatu 8, FI-33014 Tampere, Finland; † Clinical Hematology, Department of Internal Medicine, Tampere University Hospital, Medisiinarinkatu 3, FI-33520 Tampere, Finland

**Keywords:** ATP, kinase activity, kinome, nucleotide binding, pseudokinase, signalling

## Abstract

Pseudokinases are classified by the lack of one or several of the highly conserved motifs involved in nucleotide (nt) binding or catalytic activity of protein kinases (PKs). Pseudokinases represent ∼10% of the human kinome and they are found in all evolutionary classes of kinases. It has become evident that pseudokinases, which were initially considered somewhat peculiar dead kinases, are important components in several signalling cascades. Furthermore, several pseudokinases have been linked to human diseases, particularly cancer, which is raising interest for therapeutic approaches towards these proteins. The ATP-binding pocket is a well-established drug target and elucidation of the mechanism and properties of nt binding in pseudokinases is of significant interest and importance. Recent studies have demonstrated that members of the pseudokinase family are very diverse in structure as well as in their ability and mechanism to bind nts or perform phosphoryl transfer reactions. This diversity also precludes prediction of pseudokinase function, or the importance of nt binding for said function, based on primary sequence alone. Currently available data indicate that ∼40% of pseudokinases are able to bind nts, whereas only few are able to catalyse occasional phosphoryl transfer. Pseudokinases employ diverse mechanisms to bind nts, which usually occurs at low, but physiological, affinity. ATP binding serves often a structural role but in most cases the functional roles are not precisely known. In the present review, we discuss the various mechanisms that pseudokinases employ for nt binding and how this often low-affinity binding can be accurately analysed.

## INTRODUCTION

**Figure 1:**
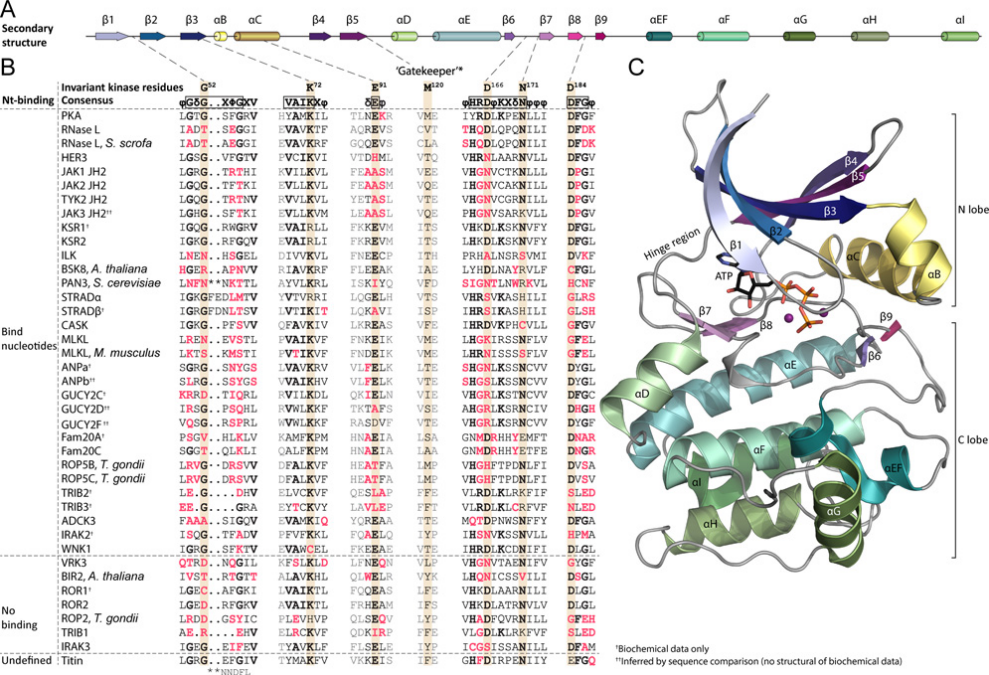
Conserved motifs and residues contributing to nt binding and kinase activity in (pseudo)kinases (**A**) Schematic depiction of the secondary structure elements of ePKs. Relative sizes and positions of elements are based on PKA (PDB: 4WB5). (**B**) Sequence alignment of selected pseudokinases and PKL proteins classified based on current information on nt binding. Conserved PK regions relevant to nt binding are shown. The six highly conserved residues contributing directly to nt binding or catalytic activity are highlighted. *The gatekeeper residue is not part of the 10 conserved kinase residues identified in [14]. All sequences represent human proteins unless otherwise noted. The sequence alignment was made using Clustal W [137,138] and manually corrected based on crystal structures and previous alignments [32,92,139] where available. For non-ePKs the sequences shown are the (predicted) functional/structural equivalents of the conserved residue in question (secondary structure of e.g. ADCK3 or Fam20 kinases is different from the one shown in **A**). Fam20C is included as an example of an active Fam20 kinase. (**C**) 3D structure of human PKA (PDB: 4WB5) shown as an example of an archetypal ePK. ATP is shown in sticks and the two magnesium cations as purple spheres. Colours of secondary structure elements are as in (**A**).

In their landmark paper in 2002, Manning and colleagues presented for the first time a comprehensive catalogue of human protein kinases (PKs) [1]. One of the salient findings of their analysis was that ∼10% of the 518 human PKs lack at least one of the classical conserved catalytic residues described by Hanks et al. [2]: the β3 lysine (K in the ‘VAIK’ consensus motif, Figure 1), the catalytic aspartate (D in ‘HRD’) or the cation-binding aspartate (D in ‘DFG’). Some of these kinases, like the WNK (‘With no lysine’) family, were already known to have phosphoryl transfer activity despite the lack of canonical catalytic sites. Yet, the other 50 identified proteins or protein domains were deemed likely to be inactive and consequently dubbed ‘pseudokinases’. Subsequently, also some of these pseudokinases (like Haspin) were shown to be catalytically active, and thus could be reclassified as atypical PKs [3]. For these and some more recent examples of catalytic activity like KSR2 [4], HER3 [5], JAK2 JH2 [6] or CASK [7] the distinction of pseudokinase and atypical kinase has become somewhat unclear. However, as proposed by Eyers and Murphy [8], the bioinformatic definition of a pseudokinase (with the inclusion of kinases that have experimentally been found to be inactive) should be maintained for the sake of clarity, and will be used in the present review as well.

Pseudokinases are spread throughout all PK families [1], indicating that at least most of them have emerged repeatedly from active kinases throughout evolution. Despite the catalytically inactive nature of most pseudokinases studied thus far, a recent survey found that 13 out of the 31 diverse pseudokinases studied are still able to bind nts [9]. Further studies on the characteristics and functions of nt binding in pseudokinases have shed light on this unexpected finding, and shown that pseudokinases employ diverse mechanisms to bind ATP and suggest that nt binding can play an important structural and functional role.

Several excellent reviews on the advances in the pseudokinase field have been written [3,8,10], including the potential importance of pseudokinases in human disease [11–13]. Recent mounting evidence of the importance of the nt-binding site of pseudokinases in particular, however, warrants a focused discussion on this aspect. The present review aims to provide such a comprehensive update on the heterogeneity of nt binding in pseudokinases.

## MOTIFS INVOLVED IN NT BINDING IN PK DOMAINS

Pseudokinases belong to the protein kinase-like (PKL) superfamily which share a conserved fold with an N lobe comprised mostly of β strands and a mostly α-helical C lobe (Figure 1C). The catalytic mechanism in PKL proteins involves 10 residues [14] that are highly conserved even if the proteins may otherwise lack sequence conservation [14,15]. Six out of these 10 residues are directly involved in nt or substrate binding or catalysis [14] (highlighted in Figure 1B), whereas the function of the remaining four residues is still not completely understood [14], but is probably mainly structural [16].

The first of the critical residues for nt binding is Gly^52^ (as the *de facto* archetypal protein kinase, human cAMP-dependent protein kinase A (PKA) is usually used for numbering of amino acid residues in protein kinase motifs and will also be used in the present review) in the glycine-rich loop (‘Gly-rich loop’, also known as the ‘phosphate-binding loop’ or ‘P-loop’) located between strands β1 and β2 (Figure 1). The function of the Gly-rich loop is best understood in PKA, in which glycine at this position enables the peptide backbone of the tip of the loop (Ser^53^) to bind the γ-phosphate of ATP. Mutation of the Gly-rich loop, and especially Gly^52^, lowers affinity towards ATP and affects kinase activity [17] as the γ-phosphate can no longer be efficiently positioned for phosphoryl transfer between the tip of the Gly-rich loop and a basic residue (Lys^168^) from the so-called ‘catalytic loop’ situated between β6 and β7 (Figure 1) [18]. Although the function of the Gly-rich loop is well known for PKA, the motif has not been extensively studied in other kinases and it is unclear how universal this function is.

**Figure 2:**
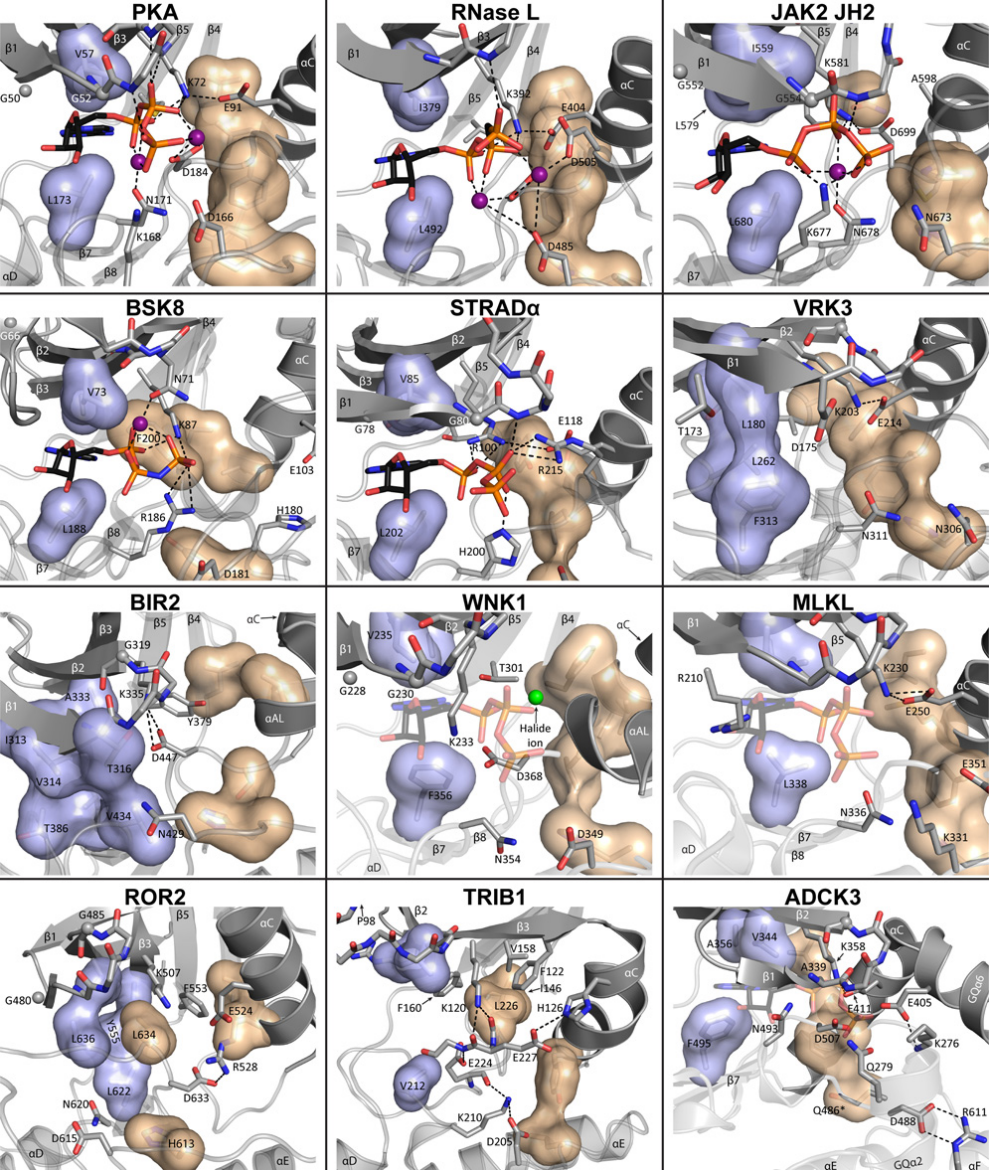
Diverse ATP-binding pockets and nt-binding modes among pseudokinases Crystal structures of ATP-binding pockets of selected representative (pseudo)kinases and PKL proteins with varying nt-binding modes. Shown are human PKA (PDB: 4WB5), human RNase L (4OAV), human JAK2 JH2 (4FVQ), *Arabidopsis thaliana* BSK8 (4I94), human STRADα (3GNI), human VRK3 (2JII), *A. thaliana* BIR2 (4L68), *Rattus norvegicus* WNK1 (4Q2A), human MLKL (4MWI), human ROR2 (4GT4), human TRIB1 (5CEM) and human ADCK3 (4PED). ATP shown in WNK1, MLKL and ADCK3 was modelled based on PKA (4WB5), as no ATP-bound structures exist, even though they verifiably bind adenine nts. ATP or ATP-analogues (e.g. AMP-PNP for BSK8) are shown as sticks with elements coloured as follows: carbon: black, oxygen: red, nitrogen: blue, phosphorus: orange. Divalent cations are shown as purple spheres. The halide ion in WNK1 is shown in green. The R spine is shown as a beige volume filling model, whereas the top of the C spine, encompassing the hydrophobic purine-binding pocket is shown in light blue. Hydrophobic side chains occluding the purine-binding pocket are shown as part of the C spine for VRK3, BIR2 and ROR2, where the pocket is occluded. Gly-rich loop glycines are shown as grey spheres with Gly-rich loop side chains omitted, unless of special note. Water molecules from the crystal structures have been omitted for clarity. *Only one possible conformation given for Gln^486, ADCK3^ is shown.

The second residue is Lys^72^ of the ‘VAIK’ motif in β3, which is the most conserved residue in all PKL proteins, and the only residue not missing in any of the known families (Figure 1) [14]. Despite its virtually universal conservation and its position next to the α and β phosphates of ATP (Figure 2, PKA), the function of Lys^72^ in nt binding is not entirely clear, and it has been reported to be dispensable for nt binding in multiple canonical kinases [19–22]. However, the lysine seems to be required for nt binding in the pseudokinases GUCY2C [23], HER3 [5], TRIB2 [24] and murine (and to a lesser extent also human) MLKL [25,26]. Lys^72^ (or another lysine in its spatial position, like Lys^223, WNK1^ in the WNK family [27]) is absolutely required for catalytic activity both in multiple canonical kinases [19,20,22], as well as in the low-activity pseudokinase JAK2 JH2 [6]. Even though its function in ATP binding is somewhat unclear, Lys^72^ is critical in making a salt bridge to the conserved Glu^91^ in the C helix (αC) (Figure 2, PKA), thus linking αC to the nt-binding pocket and the helix in the ‘in’ position, which is a hallmark of the active kinase conformation [16,28].

The last three of the six conserved residues are required for catalysis: Asp^166^ (‘HRD’) and Asn^171^ in the catalytic loop, and Asp^184^ in the ‘DFG’ motif (Figure 1). Asn^171^ and Asp^184^ participate in the binding of the two divalent cations accompanying ATP in the canonical mode of ATP binding in kinases (Figure 2, PKA). Similarly to the β3 lysine, Asp^166^ and Asp^184^ are not absolutely required for ATP binding, but rather needed for efficient catalysis [22], Asp^166^ being the catalytic base in the phosphoryl transfer reaction [29].

In addition to these conserved single residues, the purine pocket of the nt-binding site is lined with a group of hydrophobic residues from β2 (Val^57^) and β3 (Ala^70^) from the N lobe, and β7 (Leu^173^) from the C lobe (Figure 2, PKA). These residues are part of the so-called ‘catalytic spine’ (C spine), which is a conserved hydrophobic structure typically found in active kinases [16,30,31], and which is completed upon binding of a nt's purine ring between the N and C lobes (shown in light blue in Figure 2). Additionally, PKs also have another nonlinear, conserved structural element called the ‘regulatory spine’ (R spine) [16,30,31], made up of four residues from DFG, HRD, αC and the αC-β4 loop (shown in beige in Figure 2). This structure is stabilized by phosphorylation of the activation loop in canonical kinases, and a fully assembled R spine is usually a prerequisite for kinase activity [16,30,31].

### Functional prediction based on sequences–exceptions to the rule

The aforementioned conserved residues have been used to predict nt-binding ability and/or catalytic activity of unknown PKs. The presence of an intact Gly-rich loop and VAIK motifs, for example, seem to have been the best predictors for catalytic activity in the past [32]–even in noncanonical active kinases like CASK, which lacks Asp^184^ but has an intact Gly-rich loop and a β3 lysine. However, nt-binding ability and mechanisms cannot be reliably predicted from sequence data alone, as is shown by the innovative non-canonical nt-binding modes employed by several pseudokinases (see below). Thus, accurate biochemical and biophysical measurements that allow careful differentiation between nt binding, catalytic activity, and their possible physiological roles, are a necessity when analysing divergent proteins like pseudokinases.

## METHODOLOGY OF MEASURING NT BINDING

The inherent properties of pseudokinases, i.e. low or absent kinase activity coupled to unknown nt binding, are often incompatible with traditional kinase assays and often multiple methods and rigorous controls need to be employed in order to obtain reliable results. Current techniques, and their challenges with respect to pseudokinases have been recently described in the excellent review by Lucet et al. [33]. In order to provide the background for the following inspection of nt-binding properties in pseudokinases, we will highlight some important technical aspects in the present study as well.

Phosphoryl transfer or ATP hydrolysis activity of pseudokinases is generally orders of magnitude lower compared with active kinases, and therefore even tiny kinase contaminations can lead to false conclusion of pseudokinase catalytic activity [34]. As an example, in vertebrate mitotic checkpoint protein BUBR1 kinase-inactivating mutations and deletion studies demonstrated that the initially observed kinase activity was actually derived from contaminating kinases [35]. Furthermore, as with conventional kinases, choice of substrate can be critical, and the lack of phosphorylation of *a priori* likely substrate candidates or an artificial substrate does not preclude that the protein could have specific activity in the context of a physiological protein complex [8,12].

X-ray crystallography gives atomic-level information on the structure of a nt-binding pocket and can be used to predict, or in the case of nt-bound structures, unequivocally verify nt binding. KSR2 [4], HER3 [5,36], TYK2 JH2 [37], JAK2 JH2 [38], STE20-related adaptor alpha (STRADα) [39], ILK [40] and CASK [7], as well as a few other pseudokinases and PKLs from human and other species, have been crystallized in complex with nts providing solid proof and mechanistic information of nt binding (see Table 1). While X-ray crystallography is limited to determination of rigid protein structures, NMR is applicable for small proteins (*M*_r_ ≤30–40 kDa [41]) in a soluble state to reveal dynamic structures [42], and e.g. verify absence or presence of nt binding [43].

**Table 1:** Summary of published pseudokinase crystal structures Abbreviations: P-PCP, β,γ-methyleneadenosine 5′-triphosphate; AMP-PN, AMP phosphoramidate. *Crystallized with surface mutations (W659A, W777A, F794H). †Inferred from biochemical data or close homology to published structures showing the nt-binding mode. ‡Can be made cation-dependent with four point mutations [83]. ^§^Mutated as explained in [77]. ^¶^Not identical with ROP2 sequence in 2W1Z.

	Crystal structures						

Protein	PDB ID	Species	Ligand	Complex	*K*_d_ for ATP	Binding mode	Other
KSR2	2Y4I	*Homo sapiens*	ATP + Mg	MEK1	Unknown	1 cation	Active, phosphorylates MEK1 [4]
HER3	4OTW	*H. sapiens*	Bosutinib	–	10^−6^ M [5]	1 cation	Active, autophosphorylates its own intracellular region, when immobilized on vesicles [5]
	4RIW	*H. sapiens*	AMP-PNP + Mg	EGFR KD (V624R, F973A, L977A)			
	4RIX (Q709R)	*H. sapiens*	AMP-PNP + Mg	EGFR KD (V624R, F973A, L977A)			
	4RIY (E909G)	*H. sapiens*	AMP-PNP + Mg	EGFR KD (V624R, F973A, L977A)			
	3KEX	*H. sapiens*	AMP-PNP + Mg	–			
	3LMG	*H. sapiens*	AMP-PNP + Mg	–			
TYK2 JH2	3ZON	*H. sapiens*	IKK1	–	10^−5^ M [37]	1 cation	Inactive [37]
	4WOV	*H. sapiens*	BMS-066	–			
	4OLI	*H. sapiens*	Inhibitor 7012	JH2-JH1 (D1023N)			
	5C03	*H. sapiens*	ATPγS + Mg	–			
	5C01	*H. sapiens*	Pyrazine inhibitor	–			
JAK2 JH2	4FVP*	*H. sapiens*	–	–	10^−6^ M [6,45]	1 cation	Active, autophosphorylates on S523 and Y570 [6]
	4FVQ*	*H. sapiens*	ATP + Mg	–			
	4FVR (V617F)*	*H. sapiens*	ATP + Mg	–			
JAK1 JH2	4L00	*H. sapiens*	–	–	10^−6^ M [45]	1 cation^†^	Inactive [65]
	4L01 (V658F)	*H. sapiens*	–	–			
MLKL	4BTF	*Mus musculus*	–	–	10^−5^ M (TSA) [26]	No cation^†^ [9,25,26]	Inactive [25]
	4MWI	*H. sapiens*	–	–			
	4M67	*H. sapiens*	–	–			
	4M68	*M. musculus*	–	–			
	4M69	*M. musculus*	–	RIP3 KD			
STRADα	3GNI	*H. sapiens*	ATP	MO25	10^−4^–10^−6^ M [39,82]	No cation [9]	Inactive [140]
	2WTK	*H. sapiens*	AMP-PNP	MO25 + LKB1			
VRK3	2JII	*H. sapiens*	–	–	None	N/A	Inactive
ILK	3KMW	*H. sapiens*	ATP + Mg	α-Parvin	10^−6^ M [34]	1 cation	Inactive [34,40]
	3KMU	*H. sapiens*	–	α-Parvin			
	3REP	*H. sapiens*	ATP + Mn	α-Parvin			
CASK	3C0G	*H. sapiens*	3′-AMP	–	10^−3^ M [7,9]	No cation	Active, cations inhibit binding and activity. [7,24,83]
	3C0I	*H. sapiens*	3′-AMP	–			
	3C0H	*H. sapiens*	AMP-PNP (only AMP visible)	–			
	3TAC	*H. sapiens*	–	Liprin-α2			
	3MFS (4M)^‡^	*H. sapiens*	AMP-PNP	–			
	3MFU (4M)^‡^	*H. sapiens*	AMP-PNP + Mn	–			
	3MFT (4M)^‡^	*H. sapiens*	–	–			
	3MFR (4M)^‡^	*H. sapiens*	–	–			
ROR2	3ZZW	*H. sapiens*	–	–	None [55]	N/A	Inactive [55,102]
	4GT4	*H. sapiens*	–	–			
BIR2	4L68	*Arabidopsis thaliana*	–	–	None [94]	N/A	Inactive [94]
BSK8	4I92	*A. thaliana*	–	–	Unknown	1 cation	Inactive [76]
	4I93	*A. thaliana*	–	–			
	4I94	*A. thaliana*	AMP-PNP	–			
Titin	4JNW	*H. sapiens*	–	–	10^−4^ M [113]	Unknown	Disputed [112–114]
	1TKI	*H. sapiens*	–	–			
PAN3	4CYI	*Chaetomium thermophilum*	ATP + Mg	–	Unknown, but probably rather high affinity [78]	1 cation	Unknown, physiological role of PKD is to shuttle polyribonucleotides to PAN2 [81]
	4CYJ	*C. thermophilum*	ATP + Mg	PAN2			
	4CZY	*Neurospora crassa*	AMP-PNP + Mg	PAN2			
	4BWK	*N. crassa*	ATPγS	–			
	4BWX^§^	*N. crassa*	ATPγS + Mg				
	4BWP	*Drosophila melanogaster*	AMP-PN	–			
	4XR7	*Saccharomyces cerevisiae*	–	PAN2			
ROP2	2W1Z	*T. gondii*	–	–	None [43]	N/A	Inactive [43]
	3DZO^¶^	*T. gondii*	Mg	–			
ROP5B	3Q5Z	*T. gondii*	–	–	Unknown	2 cations	Inactive [110]
	3Q60	*T. gondii*	ATP + Mg	–			
	4LV5	*T. gondii*	ADP	IRGa6 (*M. musculus*)			
ROP5C	4LV8	*T. gondii*	ADP + Mg	IRGa6 (*M. musculus*)	Unknown	1 cation	Unknown
ROP8	3BYV	*T. gondii*	Mg	–	Unknown	Unknown	Unknown
RNase L	4O1O	*Sus scrofa*	–	2-5A	10^−3^ M [53]	2 cations	Inactive [52–54]
	4O1P	*S. scrofa*	AMP-PNP + Mg	2-5A			
	4OAU	*H. sapiens*	ADP + Mg	2-5A			
	4OAV	*H. sapiens*	AMP-PCP + Mg	RNA + pUp			
WNK1	4Q2A	*Rattus norvegicus*	Br	–	Unknown	Unknown	Active [124]
	3FPQ	*R. norvegicus*	–	–			
	4PWN	*H. sapiens*	–	–			
MviN	3OTV	*Mycobacterium tuberculosis*	–	–	None [47]	Unknown	Inactive [47]
	3OUK	*M. tuberculosis*	–	–			
	3OUN	*M. tuberculosis*	–	FhaA			
	3UQC	*M. tuberculosis*	–	–			
ADCK3	4PED	*H. sapiens*	–	–	Binds preferentially ADP [108]	Unknown	Inactive, can be activated with a single Gly-rich loop mutation [108]

Assays measuring nt binding are often less sensitive for low levels of contaminations than kinase assays. Isothermal titration calorimetry (ITC) and surface plasmon resonance (SPR) are quantitative, label-free methods for detection of nt binding. ITC is the only method for direct measurement of thermodynamic parameters including enthalpy, *K*_d_ and stoichiometry for ligand–protein interaction, as it directly measures the absorbed or emitted heat during a (bio)molecular interaction [34]. SPR, on the other hand, provides direct information about binding kinetics and affinity through an optical assay [37,44].

Affinity (and to a lesser extent kinetics) estimates, can also be obtained using spectrofluorometric assays using intrinsic fluorescence measurements or fluorescently tagged nts. The intrinsic fluorescence method depends on tryptophan, tyrosine and phenylalanine residue(s), whose spectroscopic characteristics change in response to nt binding, which makes these assays somewhat limited in their applicability. More widely-used fluorescence applications use fluorescent nt analogues in fluorescence spectroscopy or fluorescence polarization assays. Mant (2′(3′)-*O*-(*N*-methylanthraniloyl)) [5,6,45] and TNP (2′-3′-*O*-(2,4,6-trinitrophenyl)) [7,46] are commonly used labels, that show enhancement in fluorescence once bound to the hydrophobic ATP-binding pocket of a (pseudo)kinase. Fluorescence polarization (also known as fluorescence anisotropy) assays, using e.g. the long-wavelength fluorescently-labelled BODIPY–ATP-γ-S nt analogue, measure the increase in fluorescence polarization upon binding of a labelled small-molecule ligand to a protein. Due to its sensitivity, the assay is well-suited for screening purposes, but it has also been used to assess binding of nt analogues to pseudokinases [47]. Methods using tagged nts allow estimation of the *K*_d_ for the nt analogues, but non-specific binding is possible. Values for unlabelled nts can be estimated by their ability to displace the fluorescent analogues, which is important, as the presence of fluorescent tags can change the binding properties of a nt [39]. For competition experiments the measurement window of the assay is, however, inherently restricted by the binding affinity of the probe in question.

Binding of nts (or indeed any ligand) to proteins can also be analysed using thermal shift assays (TSA), which measure changes in thermal stability of a protein upon binding of ligands. Changes in thermal stability are measured by observing heat-induced unfolding of the protein using, e.g. intrinsic fluorescence of a protein [24,34] or extrinsic fluorescence of a dye [9] as a readout. Ligand binding generally stabilizes protein structures, and results in an increased melting temperature (*T*_m_). Correlation between *T*_m_ and *K*_d_ or IC_50_ is dependent on the protein–ligand pair in question, and TSAs should not be used as a sole method for *K*_d_ determination [48,49]. Furthermore, for low-affinity binders or cases where ligand binding does not cause large *T*_m_ shifts (Δ*T*_m_), sensitivity can be a problem.

The methods described above are primarily suited for purified recombinant proteins. Nt affinity chromatography is one of the few tools for identification of nt binders also from cell lysates. The method uses affinity beads or immobilized ATP to capture nt-binding proteins and detection by, e.g. Western blotting [23] or mass spectrometry in a high-throughput approach [50].

In summary, estimates for nt-binding affinity between different methods can vary quite significantly, and the choice of method has to be often determined empirically. In general, label-free methods, like ITC, should be favoured for accuracy and precision, but due to limitations in, e.g. protein amount, other assays are also widely used. Furthermore, the functional role and relevance of nt binding can rarely be assessed using recombinant proteins alone, and usually requires site-directed mutagenesis in cellular assays. Additionally, the modes of nt binding vary widely in pseudokinases, and they can only be revealed by solving crystal or NMR structures.

## MODES OF NT BINDING IN PSEUDOKINASES

Given the large variation of pseudokinase sequences especially at or near the nt-binding site (Figure 1), it is not surprising that pseudokinases display a wide variety of different nt-binding mechanisms. The mechanism of ATP binding is currently unknown in most pseudokinases, but structural information of 21 pseudokinases (Table 1) has shown that pseudokinases can bind ATP in a canonical fashion employing two cations or, as in most cases, in a non-canonical fashion with one or no cations visible in the crystal structure. In addition, some pseudokinases do not bind nts at all, and obtain either an active or inactive conformation by amino acid substitutions and structural rearrangements. Finally, a recent analysis of 31 pseudokinases showed that some pseudokinases were stabilized by the presence of only cations without nts [9], but the significance of this finding is currently unclear. In the present study, we provide a comprehensive structural view on nt binding in pseudokinases with a focus on the nt-binding mode in pseudokinases for which structural data are currently available.

### Bind nts

#### PKA

PKA represent the archetype for a canonical PK [51], and its mode of nt binding is the most prevalent among known PKL members. PKA binds its nt ligand ATP with two divalent cations positioned between the phosphates using the critical residues described above (Figure 2).

#### RNase L

The only known pseudokinase to date displaying a canonical nt-binding mode is the mammalian endoribonuclease RNase L. RNase L functions in the type I interferon (IFN) response where it is activated by 2′,5′-oligoadenylate (2-5A) second messengers and cleaves intracellular RNA. RNase L consists of an ankyrin repeat (ANK), a PK and a RNase domain. Despite its initial classification as an active kinase [1], the current consensus is that RNase L is an inactive pseudokinase [52–54], as it does not autophosphorylate [52], or phosphorylate the generic substrate myelin basic protein (MBP) [54]. The PK in RNase L is thought to function as a scaffold for homodimerization [54]. RNase L binds ATP with micromolar affinity and ATP binding activates RNase activity both *in vitro* and in cells, but is dispensable for RNase L dimerization in the presence of 2-5A [53]. The two recently published crystal structures of human and porcine nt-bound RNase L [53,54] show a PK fold with complete R and C spines resembling the traditional active kinase conformation (Figure 2) [31]. The structures, however, also show a non-canonical C lobe with substitutions in the activation loop and substrate recognition sites, which probably account for the inactivity of RNase L [52]. Both solved structures show a fully ordered nt in the ATP-binding pocket, which resembles RNase L's closest active homologue inositol-requiring protein 1 (IRE1). Nt binding is coordinated by conserved PK residues, except one uncommon substitution at Gly^186^ (Asp^505, RNase L^), which forms an additional interaction to one of the cations (Figure 2). The binding pocket is also flanked by the structured PK-ANK linker, which participates in 2-5A binding and interdomain interactions [53,54].

#### HER3

The most common nt-binding mode among the known pseudokinase structures is a one-cation binding mode first seen in human epidermal growth factor family pseudokinase HER3 (also known as ERBB3). As with other kinase families, pseudokinases can also be found in the group of 58 human receptor tyrosine kinases (RTKs), eight of which have been suggested to contain intracellular pseudokinase domains (PKDs) [55]. Clinically the most prominent of these is HER3, which mediates cellular signalling through ligand-induced heterodimerization with epidermal growth factor receptor (EGFR) and HER2 and allosteric activation of their kinase activity [36]. Its clinical relevance is exacerbated by the fact that HER3-mediated HER2-activation can cause resistance to HER2-targeting cancer therapies [56]. The most noticeable features of the kinase domain of HER3 are the lack of Glu^91^ (substituted by His^740, HER3^, numbering as in PDB: 4RIW) and Asp^166^ (Asn^815, HER3^), the latter of which leading to the classification of HER as a pseudokinase [1,3]. The domain is, however, able to bind ATP tightly with a *K*_d_ of ∼1 μM [5], and several crystal structures with the ATP-analogue adenylate-imidodiphosphate (AMP-PNP) have been reported (Table 1) [5,36,57], all of which show nt-bound HER3 in an inactive αC-out conformation. The nt binds together with a single Mg^2+^ cation coordinated between the AMP-PNP phosphates, Asn^171^ (Asn^820, HER3^) and Asp^184^ (Asp^833, HER3^). The conserved β3 lysine (Lys^723, HER3^) binds to both the α phosphate and Asp^833, HER3^ in a non-canonical manner most likely due to the lack of a Glu^91^-equivalent in αC. Interestingly, HER3 shows catalytic *trans-*autophosphorylation activity *in vitro*, when immobilized on vesicles at high local concentrations [5]. The biological relevance of this is uncertain, however [10,55], as the weak kinase activity has been reported to be dispensable at least for HER3-mediated ligand-dependent signalling [55].

#### JAK JH2

Janus kinases (JAKs) are non-RTKs with a rare domain architecture containing a pseudokinase (JAK homology 2, JH2) and a kinase domain (JH1) in tandem. JH2 mediates important regulatory functions and is needed both for suppression and full activation of JH1 activity [58–62]. JAKs function in a myriad of critical biological processes ranging from regulation of the immune system to haematopoiesis and metabolism [63]. The identification of multiple disease driver mutations in JAK JH2s have made them probably the most clinically studied group of pseudokinases to date. The most prominent of these mutations is the V617F mutation in JAK2 JH2 underlying multiple myeloproliferative neoplasms [64]. While JAK2 JH2 has been shown to possess low autoregulatory kinase activity [6], JAK1 and TYK2 JH2s are probably catalytically inactive pseudokinases as no autophosphorylation or kinase activity towards exogenous substrates has been detected [37,65].

Three of the four JAK JH2 domain structures have been solved over the last few years. The JH2s in all three (JAK1, JAK2, TYK2) are very similar and show a kinase domain structure with an accessible nt-binding site, a partially degraded Gly-rich loop (Gly^50^ and Gly^52^ are present, however) and an abnormally short activation loop [37,38,65]. The structures resemble the HER3 PKD in their nt-binding site architecture, as the canonical Lys^72^–Glu^91^ bond is replaced with a Lys^72^–Asp^184^ bond and an asparagine substitutes for Asp^166^ in the catalytic loop (Figure 1). In JAK2 JH2, for example, the nt phosphates coordinate around one manganese cation bound mainly by the conserved Asn^171^ (Asn^678, JAK2^). The phosphates bind between Lys^72^ (Lys^581, JAK2^), the Gly-rich loop backbone at Ser^53^ (Thr^555, JAK2^), as well as Lys^677, JAK2^, which performs the same role as Lys^168^ but is located two residues downstream along the catalytic loop (Figures 1B and 2). Lys^677, JAK2^ is a lysine or arginine in all JAK JH2s and has been shown to be essential for ATP binding in JAK2 JH2 [45]. This resembles tyrosine kinases, which use an arginine at this position to substitute for Lys^168^ [66]. The hydrophobic lining of the purine-binding pocket is conserved in all JAK JH2s, including a non-canonically large aliphatic residue in β3 (Leu^579, JAK2^).

JAK2 and JAK1 JH2’s bind ATP with micromolar affinity [6,45], whereas the ATP-binding ability of TYK2 JH2 has been somewhat unclear. In their comprehensive TSA screen of pseudokinases, Murphy et al. [9], observed a slight positive *T*_m_ shift upon addition of 200 μM ATP to TYK2 JH2, whereas Tokarski et al. [67] did not observe signs of ATP binding in their TSA or competition assays. TYK2 JH2 has, however, (along with JAK1 JH2) been found to be able to bind multiple kinase inhibitor scaffolds with submicromolar affinity [67,68]. A recent crystal structure of adenosine 5′-[γ-thio]triphosphate (ATPγS)-bound TYK2 JH2 confirms that the domain is indeed capable of binding ATP. Furthermore, MANT-ATP, SPR and TSA experiments showed that the domain binds ATP with a *K*_d_ ∼15–20 μM [37].

JAK2 JH2 has been found to possess low catalytic activity that phosphorylates two regulatory residues (S523, Y570) in JAK2 [6]. Phosphorylation of these residues facilitates the autoinhibitory interaction between JH1 and JH2 [69,70]. JAK1 and TYK2, however, do not possess kinase activity, despite binding ATP [37,65]. Comparison of the three JH2 structures shows that they all contain a helix (αAL) in the activation loop that in JAK1 and TYK2, but not in JAK2, is stabilized by two salt bridges and may block the substrate entrance [37]. Furthermore, S523 and Y570 residues are not conserved in JAK1 and TYK2 and the lack of suitable substrates may also account for the lack of phosphorylation-mediated regulation that may represent an ancestral function in JAK JH2 (JAK2 is the ancestral form).

Crystal structures of apo and ATP-bound forms do not show major binding-induced changes in TYK2 JH2 [37], and for JAK2 JH2 only a stabilization of αC has been observed [38,45]. Interestingly, however, recent mutagenesis experiments indicate that ATP-binding to JAK2 JH2 is critical for the pathogenic activation of JAK, while being largely dispensable for wild-type functions [45]. This finding, along with the recent identification of a TYK2 JH2 binding inhibitor able to modulate TYK2 activity [67] make JAK JH2s tempting pharmacological targets.

#### KSR2

Kinase suppressors of Ras (KSR) act as a dynamic protein scaffold for signalling complexes in the Ras-Raf-MEK pathway. Both KSR members, KSR1 and KSR2, have been shown to be catalytically active pseudokinases [71], with KSR2 being able to phosphorylate its binding partner MEK1 [4]. Accordingly, some of the known KSR2 loss-of-function mutations locate to the ATP-binding pocket [4]. The only published KSR structure shows ATP-bound KSR2 in complex with MEK1 (Table 1) [4]. KSR2 binds ATP with a single magnesium ion coordinated canonically by Asn^171^ (Asn^791, KSR2^) and Asp^184^ (Asp^803, KSR2^). Despite the unconventional replacement of the β3 lysine with Arg^692, KSR2^, which positions close to Asp^184^ (Asp^803, KSR2^) and is reminiscent of the Lys^72^–Asp^184^ bond in HER3 and JAK JH2s, most other typical residues in the active site are conserved with Asn^171^ (Asn^791, KSR2^) and Asp^166^ (Asp^786, KSR2^) in place (Figure 1B). As a consequence of the Lys^72^–Asp^184^ bond, αC in KSR2 is in the out position. KSR2 presents an enticing therapeutic target [13], as mutations (some of which locate to the PKD) in KSR2 affect energy balance and are associated with numerous diseases, like obesity and related metabolic problems [72].

#### ILK

Integrin-linked kinase (ILK) is an important mediator of cell motility and signal transduction by connecting integrin receptors to the actin cytoskeleton. The PKD of ILK binds ATP with micromolar affinity [34,50], and two crystal structures of ATP-bound ILK in complex with its interaction partner α-Parvin are available (Table 1). Both structures show interesting peculiarities in the ATP-binding mode of ILK. The Gly-rich loop of ILK is highly divergent, with the conserved glycines Gly^50^ and Gly^52^ substituted by Asn^200, ILK^ and Asn^202, ILK^ (Figure 1B). Interestingly, these residues make contacts with the ATP phosphates with their side chains rather than the peptide backbone [40], as is usually the case for the Gly-rich loop. Furthermore, the phosphates are also coordinated by a noncanonical lysine (Lys^341, ILK^ substituting Gly^186^ in the DFG (DVK^ILK^) motif), which lunges over in the binding site close to the γ-phosphate. The lack of Gly in the DFG motif also probably locks ILK in a ‘DFG-in’ conformation [40]. The single cation seen between the ATP phosphates is coordinated solely by Asp^184^ (Asp^339, ILK^), as other usually cation-coordinating residues are substituted by non-canonical residues (e.g. Asn^171^ by Ser^324, ILK^, Figure 1). The unusual binding mode, which includes binding of the β3 lysine to the α and γ phosphates (instead of α and β), causes ATP to adopt a binding conformation not usually seen in kinases, with the γ-phosphate pointing up towards the N lobe of the domain [40].

Functionally, ATP binding is needed for ILK to interact with its interaction partner α-Parvin [34,73], even though the observed effects of ATP on the macromolecular structure and multiple biochemical and biophysical properties are small [34]. The catalytic (in)activity of ILK has been controversial [74], but evidence from recombinant and purified ILK as well as the multiple changes in the active site residues (most critically a lack of the traditional catalytic base, Asp^166^), point towards ILK being catalytically inactive [34,40].

#### BSK8

Plants rely heavily on receptor kinases in their cellular signalling, and receptor-like kinases (RLK) constitute one of the largest gene families in plants [75]. Interestingly, a disproportionately large percentage of RLKs are predicted pseudokinases. In *Arabidopsis*, for example, ∼20% of ∼600 RLK genes, are predicted pseudokinases, whereas the same portion is only ∼13% in the whole kinome (totalling ∼1000 kinases) [75]. One of the very few plant pseudokinases that has received closer attention to date is the brassinosteroid signalling kinase 8 (BSK8) from *Arabidopsis*. Its structure was recently solved with and without AMP-PNP [76]. The structures show a kinase domain with a rather unconventional, enlarged Gly-rich loop that bends into the ATP-binding pocket when no ligand is bound, but is displaced outwards in a loop structure that makes room for a Mg^2+^ ion binding in a non-canonical manner between the phosphates of AMP-PNP and the side chain of Asn^71, BSK8^ (colocalizes structurally with Gly^52^) in the Gly-rich loop, which itself is displaced outwards from the nt-binding pocket (Figure 2). Irrespective of ligand binding, the rather short activation loop is in a somewhat closed conformation, and Phe^185^ from the DFG motif (Phe^200, BSK8^ in CFG^BSK8^) bends deep into the nt-binding pocket and partially fills out a void in the back of the pocket caused by the unusually small gatekeeper alanine (Ala^132, BSK8^). This DFG conformation is the first of its kind in PKs. Despite its capability to bind ATP, BSK is most likely an inactive kinase with no detected auto- or substrate phosphorylation activity [76]. The biological role of the unusual ATP-binding pocket is thus far unknown.

#### PAN3

Human PAB1P-dependent poly(A)-nuclease (PAN3) forms a poly(A)-specific RNase complex together with the exonuclease PAN2. The active PAN2–PAN3 complex functions in both general and miRNA-mediated mRNA deadenylation. PAN3 contains a dimerization domain and a highly divergent PKD that lacks most of the typical kinase structures, including a peptide substrate-binding site, and was not classified as a (pseudo)kinase by Manning et al., [1]. Solving of the structure of the kinase-like domain of PAN3 still showed a clear PK fold able to bind ATP [77,78]. Furthermore, an intact nt-binding site was required for activation of the mRNA-targeted exonuclease activity of PAN2 [77,79]. An explanation for this finding has recently been proposed by three independent studies [78–80], which all point towards a common model of PAN2–PAN3-mediated mRNA degradation. In the model, the highly divergent ATP-binding site of PAN3 actually functions as a polyribonucleotide-binding domain, which shuttles RNA to the exonuclease domain of PAN2 [81], thus providing a striking example of creative use of the nt-binding site of a pseudokinase scaffold.

#### STRADα

The STRADα is a pseudokinase that forms a trimeric tumour suppressor complex together with MO25α and the active protein threonine kinase LKB1 (liver kinase B1), mutations in which are linked to various cancers including the Peutz-Jeghers intestinal cancer syndrome [11]. STRADα is able to bind ATP with a micromolar *K*_d_ [39,50,82] in a cation-independent manner [39,82]. Structural studies of the STRADα–MO25 complex [39], as well as the whole ternary complex [46], showed that despite the cation-independence, STRADα binds ATP in a more or less canonical mode with Arg^215, STRADα^ from GLR^STRADα^ (DFG) and His^200, STRADα^ (Asn^171^) substituting for the cation (Figure 2) [39]. The conformation of STRADα in the complexes resembles the active conformation of kinases with an ordered activation loop and an intact R spine. This conformation enables STRADα to bind LKB1 in a traditional PK–substrate interaction. Interestingly, even though early mutagenesis data suggested that ATP binding to STRADα was not needed for its function [82], detailed analysis of the STRADα–MO25 complex in controlling LKB1 activity has since shown that ATP binding to STRADα enhances the MO25 interaction in a cooperative manner thus strengthening LKB1 activation in the STRADα–MO25α–LKB1 complex [39].

#### CASK

The calcium/calmodulin-dependent serine protein kinase (CASK) belongs to the membrane-associated guanylate kinase (MAGUK) protein family, and consists of an N-terminal Ca^2+^/calmodulin-dependent protein kinase (CaM-kinase) domain as well as L27, PDZ, SH3, and guanylate kinase domains. Its biological functions have not been completely elucidated, but it is known to be involved in synaptic processes during development and regulating cell proliferation and behavioural responses [7,11]. Although initially classified as a pseudokinase [1], the CaM-kinase domain of CASK is now most commonly classified as an atypical kinase [7,16,32,83] as it has been shown to be able to catalyse autophosphorylation and phosphorylation of its substrate Neurexin-l *in vitro* [7]. Intriguingly, the phosphoryl transfer activity of CASK is not only cation-independent, but actually inhibited by them [7]–a characteristic, which can be reversed by four point mutations in the ATP-binding pocket, which transform CASK into a Mg-sensitive kinase [83]. Wild-type CASK binds ATP weakly (*K*_d_ in millimolar range) [7,9], and in the only crystal structure of wild-type CASK with an ATP analogue (AMP-PNP) the β and γ-phosphates are disordered and not visible (Table 1). Despite its unusual binding pocket characteristics, CASK has been shown to be able to bind multiple known kinase inhibitors, including, e.g., the pan-kinase inhibitor staurosporine [68].

#### MLKL

MLKL protein is an evolutionarily interesting pseudokinase that has critical functions in necroptosis (i.e. death-receptor-initiated cell death, when execution of apoptosis is prevented), where MLKL is phosphorylated on its activation loop by the receptor-interacting serine-threonine kinase 3 (RIP3) [84]. MLKL is an extremely divergent pseudokinase with noncanonical residues in the Gly-rich loop, HRD, as well as DFG-motifs (Figure 1B). Interestingly, the Gly-rich loop and the HRD motif also differ between mouse and human MLKLs, suggesting relaxed evolutionary constraints, or very recently evolved specialized functions [26]. The crystal structures of the murine and human MLKL kinase domains have been solved independently by two groups [25,26,85], and show distinctly different conformations between the species. Human MLKL exhibits the canonical Lys^72^–Glu^91^ bond (Lys^230, MLKL, Hs^–Glu^250, MLKL, Hs^, Figure 2), yet this bond is disrupted in murine MLKL where the activation loop forms a helix in between the β3 lysine and αC [25,26,85]. Although these structures could represent two conformations of the same protein, they are also accompanied by rather significant differences in primary sequence in usually constant motifs (Figure 1B).

Both human and murine MLKL bind ATP, but only in the absence of cations, which is in line with the altered cation-binding residues in the pseudoactive site [25,26]. Of the presumed ATP-binding residues, human and murine MLKL only share Lys^72^ [25,26], and even here, the need for lysine at this position is not absolute in human MLKL, as Lys^331, MLKL, Hs^ from the HRD (HGK^MLKL, Hs^) motif can contribute to ATP binding [26]. In murine MLKL this residue is Asn^318, MLKL, Mm^, and the need for the β3 lysine for ATP binding is absolute [25].

The ATP-binding ability does not seem to be needed for the physiological role of (at least murine) MLKL, as probed with K-to-M mutations of the β3 lysine [25]. Interestingly, however, overexpression of murine MLKL pseudoactive site (K219M, Q343A) or phosphomimicking activation loop mutants (S345D) in MLKL^−/−^ cells caused cell death even without stimulation, suggesting that changes in the pseudoactive site could mimic the activation of MLKL by RIP3 [25].

#### Guanylate cyclases

Another interesting group of pseudokinase-containing proteins are the membrane guanylate cyclases [pGC for particulate guanylate cyclase (GC); five members in humans: atrial natriuretic peptide receptor type A (ANPa), atrial natriuretic peptide receptor type B (ANPb), GUCY2C, GUCY2D and GUCY2F] that produce the second messenger cyclic GMP (cGMP) from GTP. PGCs are transmembrane proteins with an extracellular ligand-binding domain and an intracellular PKD next to the catalytic GC domain. Interestingly, the PKD seems to have a functional ATP-binding site exhibiting β3 lysine-dependent ATP binding [23,86]. This binding site in the PKD was originally considered to be the allosteric site causing the well-known ATP-sensitivity of GC catalytic activity [87]. Recently, however, Robinson and Potter [88] showed that the ATP-binding site exerting the allosteric regulation site is, in fact, most likely in the catalytic domain itself. According to their model, two pGC catalytic domains form an asymmetric dimer upon activation, and the nt-binding site of one domain acts as the allosteric ATP-binding site, whereas the other acts as the catalytic site binding substrate GTP [88]. Intriguingly, this leaves the pGC PKDs with functional ATP-binding sites without a known physiological function.

#### Fam20A

A very recently identified pseudokinase, Fam20A (family with sequence similarity 20, member A), is found in the secretory pathway. Although phosphorylated secreted proteins (like casein) had been known for well over a century, the kinases responsible for these phosphorylations had remained elusive until very recently [89]. The atypical PK Fam20C was identified as the physiological kinase responsible for phosphorylating not only casein [90] but actually the vast majority of all secreted phosphoproteins in humans [91]. In some tissues, like dental tissues and the lactating mammary gland, secreted protein phosphorylation seemed to be activated by a closely related paralog, Fam20A, also implicated in biomineralization along with Fam20C [92]. Interestingly, Fam20A is a pseudokinase unable to catalyse phosphotransfer or ATP hydrolysis itself, due to the critical Asp^184^ being exchanged by Gln^258, Fam20A^ (Figure 1B) [92]. Careful examination showed that Fam20A coexpresses and directly interacts with Fam20C, resulting in activation of Fam20C catalytic activity by increasing *k*_cat_ and decreasing *K*_m_ [92]. This activation was inhibited by the presence of known Fam20A disease mutations [92], which most likely act by disrupting the structural integrity of the protein [93]. Despite lacking catalytic activity, Fam20A was found to be able to bind ATP in a TSA, and back-mutating the only non-canonical critical residue (Gln^258, Fam20A^) to a more canonical acidic residue (Glu) restored catalytic activity [92]. Simultaneously, however, the Fam20A(Q258E) mutant showed slightly reduced ability to activate Fam20C activity, which the authors speculate might be due to a lack of permanent occupation of the ATP-binding pocket of Fam20A [92]. Whether this is the case, and ATP-binding to Fam20A is critical for its ability to activate Fam20C remains to be clearly shown.

### No nt binding

#### VRK3

The vaccinia-related kinase (VRK) family consists of three vertebrate members, two of which are active kinases (VRK1–2) and one (VRK3) a pseudokinase. VRK3 was one of the first pseudokinases with a published crystal structure [32], and it showed a kinase fold locked in a rather stable ‘pseudoactive’ conformation with the N and C lobes closed, αC rotated inwards, Lys^72^–Glu^91^ (Lys^203, VRK3^–Glu^214, VRK3^) intact, and a fully formed R spine assembled (Figure 2). Strikingly, the ATP-binding site of VRK3 is inaccessible as the purine-binding pocket is filled with large hydrophobic side chains (Phe^313, VRK3^, Leu^262, VRK3^ and Leu^180, VRK3^) thus completing the C spine without a bound nt (Figure 2). Furthermore, the severely degenerated Gly-rich loop extends side chains [Thr^173, VRK3^ (Gly^50^) and Asp^175, VRK3^ (Gly^52^)] into the pocket, thus precluding nt binding completely. Correspondingly, VRK3 does not show binding of nts or nt-analogues in TSAs [9,32]. VRK3 is a prime example how sequence conservation can be ‘inverted’ in pseudokinases, as the catalytic site is very poorly conserved with many substitutions (Figure 1B), yet parts of the molecular surface are highly conserved probably representing key protein–protein interaction regions [32].

#### BIR2

A pseudokinase clearly shown to adopt an αC-out conformation in conjunction with a blocked ATP-binding pocked is BCL2-antagonist/Killer 1 (BAK1)-interacting receptor-like kinase 2 (BIR2) from *Arabidopsis*. BIR2 belongs to the leucine-rich repeat (LRR) RLKs, which comprise about one third of all *Arabidopsis* RLKs, and whose kinase domains share a common origin with the *Drosophila* Pelle and human IRAKs (interleukin-1 receptor-associated kinases) [94]. The crystal structure of the BIR2 cytosolic portion shows a kinase domain with a mostly occluded nt-binding site. This is mainly caused by a downward shifted conformation of the Gly-rich loop backbone as well as bulky side chains protruding into the binding site (e.g. Ile^313, BIR2^, Val^314, BIR2^, Thr^316, BIR2^ corresponding to Leu^49^, Gly^50^ and Gly^52^). These side chains form a rigid hydrophobic network extending all the way to the catalytic loop Val^434, BIR2^ (Asn^171^) (Figure 2), thus precluding binding of nts or nt analogues, as also verified with NMR [94]. αC of BIR2 is in the typical ‘out’ position, with a short helix (αAL in Figure 2) formed from the activation loop after the DFG motif (DSG^BIR2^) taking position between αC and the occluded nt-binding site. This conformation is further strengthened by a Lys^72^–Asp^184^ salt bridge (Lys^335, BIR2^–Asp^447, BIR2^). The blocked nt-binding site together with the inactive conformation make BIR2 probably a catalytically inactive pseudokinase, which interacts constitutively with its target of regulation, BAK1 [94]. The large amount of BAK1 phosphorylation sites on BIR2 have brought forth the hypothesis that BIR2 might regulate BAK1 by acting as a ‘dummy substrate’ for it [94].

#### ROR2

In addition to HER3, there is currently only one other published structure of a human RTK PKD: ROR2, or receptor tyrosine kinase-like orphan receptor 2. Despite its name, the ROR family (comprising ROR1 and ROR2 in vertebrates) is now known to function as receptors for several Wnt ligands [95]. ROR2’s biological functions are mainly in early embryonic development, although recent evidence has shown ROR2 expression to be up-regulated in many cancers, making ROR2 a potential drug target [96]. Catalytic activity of ROR2 is controversial [55,96]. Both ROR1 and ROR2 were predicted to be active [1] as the primary sequences include all catalytic residues (with the exception of Gly^52^, Figure 1B), and even two Tyr residues in the activation loop. Also, numerous groups have reported (auto)phosphorylation of ROR2 from immunoprecipitated extracts [97–101]. However, whether the apparent catalytic activity stems from interacting kinases or ROR2 itself is in most cases unclear [96]. Efforts using purified recombinant ROR1 and ROR2 cytoplasmic portions, including the kinase domains, have thus far been unable to detect catalytic activity [102] or nt binding [55].

The two published crystal structures of the ROR2 kinase domain (Table 1) show the domain in an inactive, DFG-out, conformation which resembles the inactive conformation of IRK (insulin receptor kinase) [103], where the activation loop bends into the nt-binding pocket occupying the position of the nt phosphates (Figure 2) [104]. However, the inactive ROR2 conformation differs from the canonical IRK-like conformation in three ways. Firstly, the adenine-binding pocket is occluded by Tyr^555, ROR2^ from the hinge region, which adopts an uncommon conformation (Figure 2), possibly making up for the unusually small Leu^634, ROR2^ (Phe^185^) from the DLG (DFG) motif in blocking the adenine pocket [55,104]. Secondly, Leu^636, ROR2^ blocks the phosphate-binding pocket causing Asp^633, ROR2^ (Asp^184^) to move closer to αC and thus (thirdly) enabling a direct salt bridge link to an Arg residue in αC (Arg^528, ROR2^), thus presumably further stabilizing the inactive conformation [104]. Although the DFG-αC link is not observed in all solved ROR2 structures, the first two are most likely sufficient to block out nts and explain the lack of binding [55]. Interestingly, however, the prominent role of the unusual conformation of Tyr^555, ROR2^ in occluding the nt-binding pocket has been suggested to potentially enable activation of ROR2 via phosphorylation of this residue [55,104]. This hypothesis remains yet to be tested, however.

### Variable binding modes and abilities, or nt binding undefined

#### TRIBs

There are four mammalian pseudokinases related to the *Drosophila* Tribbles: TRIB1–3 and SgK495 [24]. All TRIBs are linked to various forms of cancer [11], and they represent an interesting group of pseudokinases with a myriad of implicated biological functions [24,105,106], and possible therapeutic potential [13]. TRIBs 1–3 consist of variable N-terminal extensions (NTEs), a highly conserved PKD and a C-terminal extension including a conserved region for binding the E3 ligase constitutive photomorphogenesis protein 1 (COP1) [105]. This COP1-binding domain seems critical for the physiological function of TRIBs, and the current model is that TRIBs act as scaffolds recruiting target proteins using their PKD, and linking them with COP1 for ubiquitination [105,107]. TRIB pseudokinases have degraded acidic motifs in place of the Gly-rich loop [24], and highly noncanonical deviations in the DFG motif (e.g. SLE^TRIB1 and TRIB2^, Figure 1B). The drastic structural consequences of these changes were revealed recently by Murphy et al. [105], who managed to solve the crystal structure of human TRIB1 thus generating the first structure of a Tribbles protein. The structure shows a kinase-like fold with a traditional C lobe, but a highly altered N lobe. The degraded Gly-rich loop is short and contains a bend-inducing proline (Pro^98, TRIB1^) causing the whole loop (normally forming the top of the nt-binding pocket) to be in a retracted position. TRIB1 also exhibits a shortened αC along with a practically non-existent β3-αC loop, which locks αC into a distorted, bent conformation (Figure 2) [105]. Additionally, the noncanonical DFG motif (SLE^TRIB1^, Figure 1B) is in a conformation resembling the ‘DFG-out’ state [28] (Figure 2). This unusual configuration seems to be stabilized by multiple salt bridges, including the β3 lysine (Lys^120, TRIB1^) connecting to the Leu^226, TRIB1^ backbone (there is no Glu^91^ homologue in αC) and Lys^210, TRIB1^ (Asn^171^) connecting to Glu^224, TRIB1^, which precedes the SLE motif and is unique among human kinases [105]. Lys^210, TRIB1^ is itself stabilized by the catalytic aspartate Asp^205, TRIB1^ (Asp^166^). All of these factors combine to form a wide, yet obstructed, ATP-binding pocket incompatible with nt binding–a conclusion backed up by TSA measurements showing no observable nt binding for TRIB1 [105].

Despite sharing over >70% amino acid sequence identity with TRIB1, TRIB2 has, in contrast, been shown to exhibit significant *T*_m_ shifts in intrinsic fluorescence and TSA at 1 mM ATP in the absence of cations [24], and slight *T*_m_ shifts at 200 μM ATP [9], indicative of relatively weak ATP binding [24]. Furthermore, TRIB2 and TRIB3 have both been reported to show low cation-independent autophosphorylation activity, but the biological significance of this is unclear [24]. Murphy et al. [105] speculate that this difference could be due to a critical alteration (Tyr^134, TRIB1^ is Cys^104, TRIB2^) in the TRIB2 C helix that could enable the SLE^TRIB1 and TRIB2^ motif to acquire a more DFG-in-like state, thus enabling nt binding. Interestingly, mutagenesis experiments in cells have shown that an intact ATP-binding pocket is needed for the transforming ability of TRB2 [107].

#### ADCK3

Most of PK research has focused on eukaryotic protein kinases (ePKs), and approximately half of the 20 PKL families are yet to be structurally explored [14]. One of those families, the evolutionarily ancient UbiB family, recently had its first protein structure solved, with the crystal structure of human aarF domain-containing protein kinase (ADCK)3 [108], a mitochondrial protein involved in isoprenoid lipid metabolism. The human ADCK family (comprising ADCK1–5) had been classified as an ‘atypical kinase’ [1], but experimental validation of enzyme activity remained elusive [108]. The structure shows a conserved core PK fold with a C lobe insert and two additions to the N lobe from conserved UbiB-specific features: an NTE before β1 (helices GQα1–4), and an insert between β3 and αC (GQα5–6, Figure 2). UbiB proteins also share a striking alteration in the Gly-rich loop, which is replaced by an Ala-rich loop. The NTE and the N-lobe insertion both form α helices that fold on to the Ala-rich loop and peptide substrate-binding cleft. Specifically, a conserved KxGQ motif from the NTE and a conserved ExD motif (Glu^405, ADCK3^) from the N lobe insertion fold into the active site of the protein (Figure 2) leaving the substrate-binding pocket completely occluded. Also, catalytic activity seems to be further prohibited as the catalytic Asp^166^ (Asp^488, ADCK3^) points away from the active site towards αF, where it makes a double salt bridge to Arg^611, ADCK3^ (Figure 2)–a residue strictly conserved as either arginine or lysine in the UbiB family [108]. Despite these alterations, however, all traditional catalytic residues are present (Figure 1B), the Lys^72^–Glu^91^ (Lys^358, ADCK3^–Glu^411, ADCK3^) salt bridge is visible, and the regulatory spine is intact. Furthermore, Stefely and colleagues could show that ADCK3 was stabilized by nts in a TSA, but with a striking preference towards ADP over ATP [108]. Careful mutagenesis studies further showed that this preference could be reversed by a single mutation restoring Gly^52^ (Ala^339, ADCK3^) to the Ala-rich loop [108]. Additionally the A339G^ADCK3^ mutation also enabled Mg-ATP-mediated autophosphorylation, which was enhanced by removal of the KxGQ motif from the NTE, clearly demonstrating that the domain has catalytic potential, but is inhibited by its preference for ADP over ATP and the inhibitory insertion into the active site. Intriguingly, these regions inhibiting kinase activity were also critical for the physiological function of the yeast ADCK3 homologue Coq8p *in vivo* [108]. Whether this means that ADCK3 is truly a pseudokinase, or whether it is a PK that is activated through interactions with other proteins is currently unclear. Stefely and colleagues put forward a further hypothesis, proposing that ADCK3 would not be a PK after all, but rather could be able to phosphorylate small molecules, like lipids, which might still be able to bind to the substrate-binding pocket despite the NTE [108].

#### ROPKs

An interesting group of pseudokinases outside of vertebrates is the rhoptry kinase (ROPK) family of apicomplexans–a phylum of parasitous protists including, e.g. *Toxoplasma gondii*, in which roughly one third of all kinases are ROPKs. Many ROPKs are key virulence factors and they are exported into the parasite's host cell during infection, where they assist in transforming the host cell environment to a more permissive state for the parasite [109]. Roughly half of the 42 known ROPK subfamilies are pseudokinases [109], and ROPK genes are often found in expanded loci that are likely sites of local tandem duplication with high sequence polymorphism [110]. Furthermore, members of ROPK subfamilies probably acquired the inactivating changes individually, thus repeatedly generating new and varying pseudokinases [109]. Thus far, four ROP pseudokinase structures have been published (Table 1): ROP2 [43], ROP8 [111], ROP5B [110] and ROP5C (PDB: 4LV8, Reese M.L., Boothroyd J.C., unpublished work). All ROPKs have a common NTE subdomain, which loops around the N lobe and down next to the hinge towards the C lobe. This extension has been hypothesized to be a regulatory region phosphorylated by active ROPKs, such as the key virulence factor ROP18 [111]. In the case of ROP2 and ROP8, the NTE runs over the nt-binding site inserting a large side chain (Trp^215, ROP2^ [43], Arg^228, ROP8^ [111]) into the site, thus precluding ATP binding. Accordingly, most of the residues in and around the nt-binding site are highly variable among a subset of ROP2-like kinases (including ROP8), suggesting lack of evolutionary pressure as can be expected for a catalytically inactive pseudokinase [43]. Interestingly, however, there are still some sequence motifs in the pseudoactive sites of ROPKs, which many pseudokinases have converged to. Namely, a change of the HRD motif to HGX, where X is often a basic residue (e.g. HGH^ROP5B and C^) [110]. Similar converging substitutions are also seen in the human kinome, where 19 out of ∼50 human pseudokinases have the R-to-G substitution, whereas it is seen in only 8 out of the ∼500 active human kinases [110].

Despite changes in the nt-binding region, the peptide-binding site of ROP2 and ROP8 seem rather conserved, suggesting that these proteins might still be capable of kinase–substrate-like interactions [43]. As another example of the extensive heterogeneity among ROPKs is ROP5, which, although able to bind ATP, is probably inactive [110]. Structural analysis of ROP5B revealed a noncanonical mode of ATP binding with two magnesium ions unusually coordinated between the ATP phosphates. Despite the conservation of Asn^171^ (Asn^394, ROP5B^) the residue next to it (Asp^393, ROP5B^) actually takes its place in coordinating the ions. Furthermore, Asp^184^ (Asp^407, ROP5B^) is shifted towards αC and only coordinates one of the bound Mg ions compared with the two in the canonical mode of binding. Also, a basic side chain connecting the γ-phosphate usually provided from the catalytic loop (Lys^168^) seems to be substituted for by Arg^245, ROP5B^ from the Gly-rich loop in ROP5B [110]. ROP5B also shows other features more commonly seen in other pseudokinases, like the lack of a correctly oriented Glu^91^ analogue, and thus a possible salt bridge between the equivalents of Lys^72^ and Asp^184^ (as seen in the JAK JH2s, HER3 and KSR2).

#### Titin

An example of a protein where the kinase/pseudokinase debate has taken a recent turn, is the giant myofilament protein titin (*M*_r_ up to 4.2 MDa) found in sarcomeres. Titin contains over 200 immunoglobulin- or fibronectin type-III-like domains (actual numbers depend on the isoform in question) as well as a kinase domain (titin kinase, TK) near its C-terminus. TK consists of a mostly canonical kinase domain with a C-terminal insertion folding into its active site thus effectively blocking potential activity [112]. Early results suggested that TK could be activated by removal of the blocking C-terminal extension and simultaneous tyrosine phosphorylation of the activation loop, and that TK could phosphorylate telethonin/Tcap [112]. More recent single-molecule experiments indicated, that TK was able to bind ATP (rather weakly, *K*_d_ ∼350 μM), but only when mechanically stretched, possibly due to removal of the C-terminal extension from the active site [113]. Titin's position as a mechanosensitive, active kinase has, however, been recently questioned, as the telethonin/Tcap-phosphorylating kinase activity in insect cell preparations was shown to be likely due to a contaminating kinase [114], as bacterially produced TK lacks activity [113,114], and the telethonin/Tcap-phosphorylating kinase activity does not strictly copurify with recombinant TK [114]. Furthermore, two noncanonical substitutions in TK's VAIK and DFG motifs (Y/FMAK and EFG, respectively in vertebrate titin, Figure 1B) have been suggested to preclude kinase activity [114]. This example highlights some of the technical challenges in atypical kinase and pseudokinase research, as choice of experimental system and purity of preparations are critical determinants for the conclusions about potential activity.

#### IRAKs

IRAKs are a family of serine/threonine kinases with two active kinases (IRAK1 and 4) and two, likely inactive, pseudokinases (IRAK2 and 3). IRAK2 functions in the sustained activation of the Toll-like receptor (TLR) pathway [115], and has been implicated in colorectal cancer [116] and in the unfolded protein response [117]. IRAK2 shows cation-dependent nt binding in TSA [9], yet the molecular details of this are still unclear due to a lack of crystal structures. IRAK2 was shown to have autophosphorylation activity when immunoprecipitated from activated macrophages, raising the question of atypical kinase activity despite noncanonical kinase motifs (Gly^52^, Asp^166^ and Asp^184^ missing) [115]. The kinase activity could, however, only be observed in macrophages also expressing IRAK4 [115], suggesting that the activity observed was derived from IRAK4, as IRAK2 and IRAK4 interact *in vivo* [118]. This would be in line with early results reporting negligible autophosphorylation activity for recombinant IRAK2 [119].

Whereas IRAKs 1, 2 and 4 are active components of the proinflammatory TLR and IL-1R family signalling pathways, IRAK3 (also called IRAK-M) functions as a key modulator of inflammatory responses. The expression of IRAK3 is regulated by the other IRAK members in a negative feedback loop, where IRAK3 associates with the same signalling molecules as IRAK1, 2 and 4 and negatively regulates the pathways [120–122]. Furthermore, IRAK3 expression is up-regulated by glucocorticoid receptor activation, and has been shown to play a key role in the immunosuppressive action of glucocorticoids [123]. Recent evidence has also identified a role for IRAK3 in proinflammatory signalling [122]. The kinase domain of IRAK3 does not show nt binding in TSA [9], but it evidently has an accessible binding pocket, as it binds a number of known kinase inhibitors [9,68]. IRAK3 completely lacks an HRD motif (CGS^IRAK3^, Figure 1B) and is most likely an inactive pseudokinase [119], which exerts its function through non-catalytic mechanisms [121,122].

### Alternative catalytic mechanism

#### WNK, PRPK and Haspin

Some kinases, originally classified as pseudokinases, have later been found to possess activity via atypical mechanisms. Although this activity is often low and only detectable in *in vitro* settings, biologically significant kinase activity has been established for a number of PKs initially thought to be inactive. The most prominent example of these is WNK, which is an active kinase where Lys^72^ is substituted with a second lysine (Lys^233, WNK1^) from β2 [27,124]. Interestingly, the unusual position of Lys^233, WNK1^ has been proposed to enable WNK1 to bind a halide ion (e.g. Cl^−^) at the back of its nt-binding pocket (Figure 2) [125]. Biochemical data further suggest that when this ion is present, activation of WNK1 by autophosphorylation is inhibited, essentially making WNK1 a chloride sensor. This could explain the apparent regulation by chloride ions observed for WNK1’s downstream targets: sodium-coupled chloride cotransporters (NCCs) and potassium-coupled chloride cotransporters (KCCs) [125].

Similarly, p53-related protein kinase (PRPK), a human homologue of yeast Bud32, is catalytically active [126] despite lacking the typical β3 lysine and phosphorylates its only substrate p53 when pre-phosphorylated by Akt [127]. Another noteworthy atypical kinase is Haspin, which belongs to its own PKL family outside of ePKs [14]. Despite having a modified DFG motif (DYT^Haspin^) Haspin is an active kinase [1], contains practically all catalytically critical residues, and binds ATP in a mostly classical manner [128].

## CONCLUSIONS AND PERSPECTIVES

Most pseudokinases have evolved from active kinases by obtaining regulatory functions where catalytic function is dispensable. However, a significant proportion of pseudokinases have retained ATP-binding ability. Presently, detailed and structural information exists for only a part of these proteins (Table 1), but mounting evidence suggests a structurally and functionally important function for nt binding in several pseudokinases. Indeed, noncatalytic occupation of the nt-binding pocket is known to regulate multiple active kinases [129–131], and as pseudokinases share the same protein fold, similar functional significance for ATP binding can be expected.

As a structural cofactor, nt binding seems to regulate or enable acquisition of certain conformations in multiple pseudokinases, e.g. allowing the domain to adopt a pseudoactive conformation (like STRADα) by completing the C spine and stabilizing the domain. Other pseudokinases, like VRK3 or BIR2 (from *Arabidopsis*), have given up the need for bound nts altogether by filling out the nt-binding pocket constitutively with bulky amino acid substitutions (Figure 2). Completion of the spines (R and C), which is usually predictive of catalytic activity in canonical kinases [16,30,31], thus cannot be used as a structural predictor of catalytic activity in pseudokinases, but is still useful in describing a conformation adopted by a pseudokinase. Furthermore, most pseudokinases lack the main mode of regulation for active ePKs, namely a phosphorylated activation loop. Indeed, activation loops in many pseudokinases are very short to the point of being practically non-existent. In such an environment, structural importance of nt-binding pocket occupation in regulation or modulation of protein–protein interactions could be further increased.

The binding affinity for ATP is rather low in many pseudokinases, which poses technical challenges. However, cellular ATP concentrations are in the millimolar range [132], so nt binding should still occur in a physiological milieu even at relatively low affinities. Some very low affinity ATP binders (e.g. TRIB2, CASK) have even been hypothesized to function as cellular nt concentration sensors [24].

Functional ATP-binding pockets are required for the physiological function of multiple pseudokinases, even in the absence of catalytic activity. The detailed structural consequences of nt occupation, however, are in many cases not obvious even when using multiple biophysical methods (e.g. ILK [34]) or when crystallographic structural data with and without nt are available (see, e.g. JAK2 JH2 [45]). In these cases methods able to detect subtle changes in protein dynamics (e.g. NMR or computational methods) could provide new insights. Nevertheless, in most cases structure-guided, targeted, carefully controlled mutagenesis studies, complemented where possible with a chemical genetics approach [133], are the key to answering questions of importance of nt-binding pocket occupation.

Pseudokinases play an important regulatory role in cellular signalling, and abnormal function of several human pseudokinases has been associated with human diseases [12]. The most prominent examples of this are probably the JAK2 JH2 V617F mutation underlying multiple myeloproliferative neoplasms [64], and HER3 overexpression in many cancers [134]. In total, over 60 mutations in the human pseudokinome have been shown to cause, or be linked to, various malignancies [11–13]. With its direct link to pseudokinase regulatory functions, the nt-binding site of many pseudokinases poses an interesting option for pharmacological interventions. For example, the E351K mutation in MLKL increases affinity for ATP and is associated with human lung carcinoma [26], and mutations in the ATP-binding pocket of JAK JH2 revert the hyperactivation of pathogenic JAK JH2 mutants [45]. To date, only a few pseudokinases, namely HER3 [135], MLKL [136] and TYK2 [67], have been attempted to target pharmacologically, providing important proof-of-principle results that targeting pseudokinases could be a viable therapeutic option. The results of pharmacological targeting of MLKL and TYK2 JH2 are significant and encouraging, but it should be kept in mind that the functions of pseudokinases are very diverse and not as directly predictable as for active kinases. Further detailed analysis is required to understand the physiological functions of different pseudokinases, and insights thus gained will lay the basis for potential future therapeutic targeting attempts.

## Abbreviations

ADCK: aarF domain-containing protein kinase
AMP-PNP: adenylate-imidodiphosphate
ANK: ankyrin repeat domain (in RNase L)
ANPa/ANPb: atrial natriuretic peptide receptor type A/B, also known as atrial natriuretic peptide receptor 1 (NPR1) and NPR2, respectively. Also known as GC-A/GC-B
ATPγS: adenosine 5′-[γ-thio]triphosphate
BAK1: BCL2-antagonist/Killer 1
BIR2: BAK1-interacting receptor-like kinase 2
BSK8: brassinosteroid signalling kinase 8
CaM-kinase: Ca^2+^/calmodulin-dependent protein kinase
CASK: calcium/calmodulin-dependent serine protein kinase
COP1: constitutive photomorphogenesis protein 1
EGFR: epidermal growth factor receptor
ePK: eukaryotic protein kinase
GC: guanylate cyclase
GUCY2: guanylate cyclase, e.g. GUCY2C, also known as GC-C, and heat stable enterotoxin receptor (HSER)
HER3: human epidermal growth factor 3, also known as ERBB3
IRAK: interleukin-1 receptor-associated kinase
ITC: isothermal titration calorimetry
JAK: Janus kinase
JH: JAK homology
LKB1: liver kinase B1
MLKL: mixed lineage kinase domain-like
nt: nucleotide
NTE: N-terminal extension
PAN3: PAB1P-dependent poly(A)-nuclease
PK: protein kinase
PDZ: protein domain found in PSD95, Dlg1 and zo-1
PKA: cAMP-dependent protein kinase A
PKD: pseudokinase domain
PKL: protein kinase-like
RIP3: receptor-interacting serine-threonine kinase 3
RLK: receptor-like kinase
ROPK: rhoptry kinase
ROR: receptor tyrosine kinase-like orphan receptor
RTK: receptor tyrosine kinase
SH3: Src-homology 3 domain
SPR: surface plasmon resonance
STRAD: Ste20-related adaptor
TK: titin kinase
TLR: Toll-like receptor
TSA: thermal shift assay
VRK: vaccinia-related kinase
WNK: With no lysine
